# Atypical social cognition processing in bulimia nervosa: an fMRI study of patients thinking of others’ mental states

**DOI:** 10.1186/s13030-023-00297-y

**Published:** 2024-02-21

**Authors:** Rio Kamashita, Rikukage Setsu, Noriko Numata, Yasuko Koga, Michiko Nakazato, Koji Matsumoto, Hiroki Ando, Yoshitada Masuda, Sertap Maral, Eiji Shimizu, Yoshiyuki Hirano

**Affiliations:** 1 Research Center for Child Mental Development, Chiba University, Chiba, Japan; 2 United Graduate School of Child Development, Osaka University, Kanazawa University, Hamamatsu University School of Medicine, Chiba University and University of Fukui, Suita, Japan; 3 Department of Cognitive Behavioral Physiology, Graduate School of Medicine, Chiba University, Chiba, Japan; 4 Sato Hospital, Nanyo, Japan; 5 Department of Clinical Psychiatry, Chiba University Hospital, Chiba, Japan; 6 International University of Health and Welfare, Narita, Department of Psychiatry, Narita, Japan; 7 Department of Radiology, Chiba University Hospital, Chiba, Japan

**Keywords:** Eating disorders, Social cognition, Bulimia nervosa, Theory of mind, Task-based fMRI

## Abstract

**Background:**

Feeding and eating disorders are severe mental disorders that gravely affect patients’ lives. In particular, patients with anorexia nervosa (AN) or bulimia nervosa (BN) appear to have poor social cognition. Many studies have shown the relationship between poor social cognition and brain responses in AN. However, few studies have examined the relationship between social cognition and BN. Therefore, we examined which brain regions impact the ability for social cognition in patients with BN.

**Methods:**

We used task-based functional magnetic resonance imaging (fMRI) to examine brain responses during a social cognition task and the Reading Mind in the Eyes Test (RMET). During the fMRI, 22 women with BN and 22 healthy women (HW) took the RMET. Participants also completed the eating disorder clinical measures Bulimic Investigatory Test, Edinburgh (BITE) and Eating Disorders Examination Questionnaire (EDE-Q), the Patient Health Questionnaire (PHQ-9) measure of depression; and the Generalized Anxiety Disorder (GAD-7) measure of anxiety.

**Results:**

No difference was observed in the RMET scores between women with BN and HW. Both groups showed activation in brain regions specific to social cognition. During the task, no differences were shown between the groups in the BOLD signal (*p* < 0.05, familywise error corrected for multiple comparisons). However, there was a tendency of more robust activation in the right angular gyrus, ventral diencephalon, thalamus proper, temporal pole, and middle temporal gyrus in BN (*p* < 0.001, uncorrected for multiple comparisons). Moreover, HW showed a positive correlation between RMET scores and the activation of two regions: medial prefrontal cortex (mPFC) and anterior cingulate cortex (ACC); however, no significant correlation was observed in women with BN.

**Conclusions:**

While activation in the mPFC and ACC positively correlated to the RMET scores in HW, no correlation was observed in BN patients. Therefore, women with BN might display modulated neural processing when thinking of others’ mental states. Further examination is needed to investigate neural processing in BN patients to better understand their social cognition abilities.

**Trial registration:**

UMIN, UMIN000010220. Registered 13 March 2013, https://rctportal.niph.go.jp/s/detail/um?trial_id=UMIN000010220

## Background

Feeding and eating disorders (EDs) are some of the most severe types of mental disorders. EDs mainly consist of anorexia nervosa (AN), bulimia nervosa (BN), and binge-eating disorder (BED) [1]. The features of AN include low body mass index (BMI), negative body image, and the drive for thinness. Binge eating is the main symptom of BN, but the desire for thinness is also present. The lifetime prevalence of AN and BN in women is up to 4 and 3%, respectively [2]. BED is a common ED that, similar to BN, is characterized by overeating but without compensatory behaviors. It is reported to be the most common ED in men, with a female-to-male ratio of 9:1 for AN and BN and 6:4 for BED [3]. EDs have some of the highest mortality rates for mental disorders. Reportedly, the standardized mortality rates of AN and BN are 5.86 and 1.93, respectively [4]. Due to suicide and physical complications from undernutrition, patients with AN have a high mortality rate. Though the mortality rate of BN is less than that of AN, BN patients face numerous difficulties.

The years lived with a disability (YLD) refers to the number of years for which a patient experiences a disability. The YLD of EDs increased between 2007 and 2017 [5], as reported by a systematic analysis spanning 195 countries and territories [6]. For example, the rate of change of the YLD of BN is 10.3%, while that of mental disorders is − 1.1%. People suffering from BN have a lower quality of life compared to people with other mental disorders [5].

According to Fairburn et al. [7], one of the core symptoms in ED is the overestimation of weight and body shape owing to a misunderstanding of social cues, which may accelerate ED symptoms. Baker et al. [8] suggested that “social cognition depends on our capacity for ‘mentalizing’, or explaining an agent’s behaviour in terms of their mental states.” Happe et al. stated that mentalization refers to the understanding of others’ mental states [9]. These social cognitive abilities, especially the ability to think about other’s mental states, have been actively studied in individuals with AN [10]. For AN, a deficit of social skills [11, 12] has been revealed. Using a video task involving social interaction between a man and woman, Brockmeyer et al. [13] reported that patients with AN experience difficulty in reading others’ emotional/mental states, such as being unable to answer “What does X feel?”, despite their intact non-emotional mental states, such as being able to determine “What does X intend?” Numerous neuroimaging techniques, such as magnetic resonance imaging, have been utilized in past studies to investigate the deficit in social skills among AN patients. In one study involving a social cognition task, a reduction of activation in the middle anterior temporal cortex and medial prefrontal cortex (mPFC) was observed in AN patients but not in a healthy control group [14]. Additionally, less activation in the two regions was correlated with AN symptoms occurring 1 year later. This research suggested that the activation of the prefrontal gyrus during social cognition tasks predicts the severity of AN.

Nonetheless, few studies have focused on the social perceptions of BN in comparison to AN. Mason et al. [15] performed a systematic review of social cognitive abilities across ED, including AN, BN, and BED, and found that numerous studies supported deficits in social cognition in patients with AN; however, despite a lack of supporting studies, some social cognition deficits appear to be present in those with BN and BED. Although a meta-analysis by Kerr-Gaffney et al. [16] concluded that AN patients experience difficulty in identifying emotions, the results for patients with BN were inconclusive due to insufficient sample sizes. Additionally, some studies other than meta-analyses have examined the social cognition of BN; however, there is currently no consensus regarding the social cognitive abilities of individuals with BN because reports on the preservation of basic social cognition [17] and impairment in facial expressions [18] provide mixed findings. Among the tasks measuring social cognition, the Reading the Mind in the Eyes Test (RMET) [19], in particular, is recognized by the National Institute of Mental Health (NIMH) as a test of social processes [20]. In a meta-analysis, patients with BN had poor RMET scores compared to controls, while AN patients scored the worst [21]. However, approximately half of the individuals diagnosed with AN have been observed to experience a transition from AN-R to disorders characterized by binge eating and purging behaviors, namely AN-BP and BN [22]. Moreover, several studies reported that the inability for mentalization and empathy is also shown in both AN and BN [23, 24]. In their review, Zanella et al. [24] stated that interpersonal and emotion regulation theories contribute to problematic eating. For example, Tuschen-Caffier et al. [25] suggested that stressful interpersonal conflicts may trigger overeating in BN. Furthermore, according to Hinrichsen et al. [26], eating and negative social cognitions in BN patients are significantly exacerbated by a combination of stress reactions and impaired emotional regulation. If a misunderstanding of social cues manifests itself in an overestimation of weight and body shape, ultimately exacerbating ED symptoms [7], then a misunderstanding of social cues may also be present in BN, where the ED pathology of overestimating weight and body shape is present. In addition, because BN patients experience interpersonal difficulties prior to the onset of BN, interpersonal difficulties maintain BN symptoms [27] and BN and social functioning are closely related, we predicted that social cognition in BN would be abnormal compared to healthy controls. Therefore, we decided to investigate social cognitive abilities in BN in this study. Although many studies have indicated that emotion dysregulation and negative social cognition in BN exacerbate binge-eating symptoms, the underlying social cognitive abilities in BN are still debated. As with AN, we expect that patients with BN also have an impaired social cognition ability based on emotional faces and display different neural processes. The RMET reflects one’s abilities related to social cognition, emotion recognition, and mentalization. This is due to its characteristic of inferring the emotional state of others. In this study, we aimed to investigate whether BN individuals exhibit any impairment in social cognitive skills by analyzing the blood-oxygenation-level dependent (BOLD) signal variations during RMET and their performance in the task.

## Methods

### Participants

We recruited 32 women with BN from the Department of Psychiatry and Neurology at Chiba University Hospital and 26 healthy women (HW) from the website of Research Center for Child Mental Development of Chiba University. Five participants (four with BN and one healthy woman) were unable to complete the psychological scales, and an additional five were excluded due to head movements exceeding 3.0 mm in the SPM realignment analysis. Furthermore, three patients and one healthy woman were removed from the analysis due to intelligence quotient (IQ) score of less than 70. We used data from 22 women with BN and 22 HW in the final analyses. Patients were diagnosed by psychiatrists at Chiba University based on the DSM-IV [28] criteria. Five patients had a comorbidity of depression. Three patients were taking selective serotonin reuptake inhibitors. None of the participants were diagnosed with alcohol abuse or dependence, substance use disorders, or a critical risk of suicide. None of the HW experienced mental illness, including EDs. All patients were Japanese women. The age range was 16-38 years (mean = 26.57, SD = 6.83 years). The study was conducted in accordance with the Helsinki Declaration and met the procedures of The Research Ethics Committee of Chiba University Hospital. Informed consent was received from the participants after we explained the details of our study verbally and in writing to them.

### Psychological scales

The Eating Disorders Examination Questionnaire (EDE-Q) [29] and Bulimic Investigatory Test, Edinburgh (BITE) [30] were used to assess the symptoms related to EDs. The EDE-Q contains a total of 28 questions, including 22 items focusing on thoughts about body weight, body shape, and degree of fear of obesity that are scored on a 7-point Likert scale (0 to 6). The remaining six items focus on the actual number of times one has overeaten or vomited. Both sets of items refer to the past 28 days. Responses obtained from the questions are divided into four subscales: Restraint, Eating Concern, Shape Concern, and Weight Concern, and the total of the four subscales divided by four represents the global score. A higher global score indicates higher eating disorder severity and a score of four or higher is within the clinical range [31]. Numerous studies have demonstrated the EDE-Q’s high reliability and validity [32–35], and it has been used worldwide to perform advanced research on EDs. The BITE questionnaire comprises 33 items. It is a screening test for bulimia nervosa developed by Henderson and Freeman [30] that can also be used to assess its severity. It consists of a Symptom Assessment Scale (BITE-SAS) (30 items) and a Severity Scale (BITE-SS) (three items). BITE-SAS has a 2-item “yes” or “no” method and BITE-SS asks about the frequency of overeating and compensatory behaviors on s 5 and 7-point Likert scales, respectively. The cutoff value is 20 points, and the score of the symptom rating scale is used as a reference. The developers of the BITE reported that data from two separate populations show adequate reliability and validity [30]. The subjective severity of psychological symptoms was measured using the Patient Health Questionnaire (PHQ-9) [36], which includes nine questions on depressive symptoms experienced in the last 2 weeks, and the Generalized Anxiety Disorder scale (GAD-7) [37], which includes seven questions assessing anxiety-related symptoms in the last 2 weeks. International studies have indicated that both the PHQ-9 and GAD-7 have adequate reliability and validity [38–41]. All the above-mentioned questionnaires can be self-administered. The ranges for each scale score are as follows: EDE-Q (0–6), BITE-SAS (0–30), BITE-SS (0–39), PHQ-9 (0–27), GAD-7 (0–21). The RMET [19], translated into Japanese by Yamada and Murai (2005), was used to investigate the ability of social cognition of both patients and HW (Autism Research Centre of the University of Cambridge, https://www.autismresearchcentre.com). It consists of 36 figures showing the eyes of men and women. There are two types of tests in the RMET. One of them is a social cognition task that requires choosing one of four words for an emotional state and matching that word with a shape. The other is a gender detection task, where participants are required to look at pictures of male and female faces and answer with their sex. The former is used for measuring social cognition, especially in autism spectrum disorder (ASD) and schizophrenia. The RMET was reported by its developer [19] to be a “measure of mentalizing.” In a review conducted in 2022 [42], the RMET was described as follows: “(i) In neurotypical individuals, RMET scores are tightly correlated with other social skills (empathy, emotional intelligence, and body language reading); (ii) The RMET assesses recognition of facial affect, but also heavily relies on receptive language skills, semantic knowledge, and memory; (iii) RMET performance is underwritten by the large-scale ensembles of neural networks inside and well-outside the social brain.” (p. 1). We used the RMET in this study to investigate the abilities of social cognition of BN patients. Moreover, IQ was measured using the short version of the Wechsler Adult Intelligence Scale, third edition (WAIS-III) [43] by estimating it from two subtests [44].

### Imaging parameters

At the Chiba University Hospital, participants underwent fMRI scanning with a 32-channel head coil (Discovery MR750 3.0 T, General Electric Healthcare, Waukesha, WI, USA).

The fMRI scan was performed using the following parameters: Echo time, 30 ms; repetition time, 2000 ms; number of slices, 36; flip angle, 76; acquisition matrix, 64 × 64; slice thickness, 3.7 mm; field of view, 24.0 cm; and bandwidth, 111.11 kHz. In contrast, the 3D T1 weighted images were acquired using the following parameters: echo time, 3.184 ms; repetition time, 8.124 ms, flip angle, 15°; acquisition matrix, 256 × 256; slice thickness, 1 mm; field of view, 25.6 cm × 25.6 cm; number of excitations, 1; bandwidth, 15.625 kHz; inversion time, 420 ms; and acceleration factor, 2.

### Task-based fMRI

We set the social cognition task as the main stimulus and the gender detection task as the control task in the RMET. Moreover, we used part of the RMET in the fMRI task to better identify BOLD signal changes that occurred in response to the task by employing more challenging items. The task fMRI study by Rolls et al. [45] showed that changes in the BOLD signal during the task were larger for the more difficult task. Therefore, in our study, we reduced the number of RMET items and measured task fMRI to better capture the characteristic BOLD signal in BN. To select the items, RMET was performed for 20 individuals who were in good health. Following data analysis, 16 items were selected in order of lowest percentage of correct responses. Then, the correct answer and the most chosen answer were adopted as the two choices in the social cognition task.

**Fig. 1:**
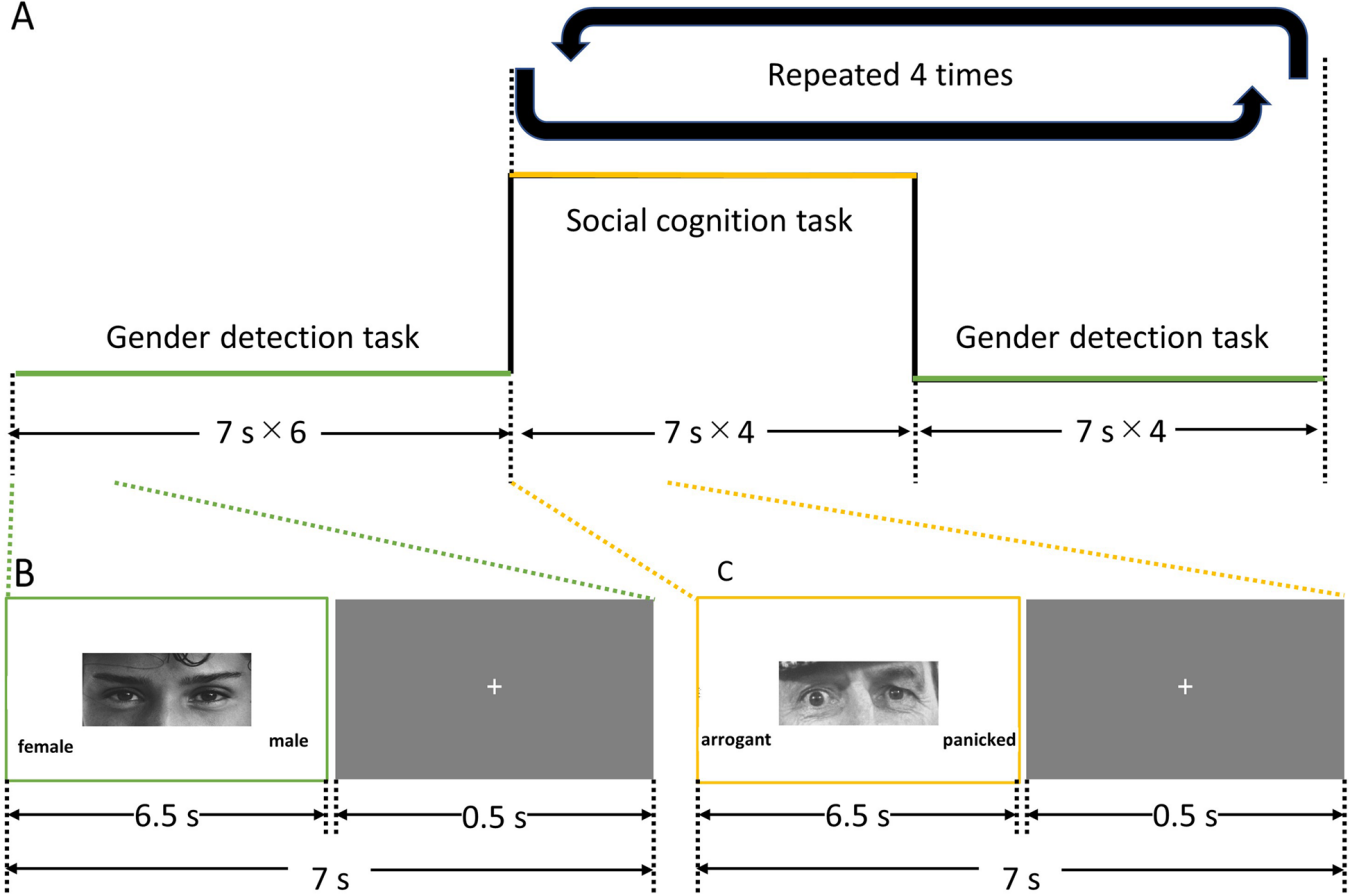
Protocol of the MRI task (**a**) The procedure of the tasks in the MRI. (**b**) The gender detection task from the “Reading the Mind in the Eyes Test (RMET)” [19], translated into Japanese by Yamada & Murai in 2005. The left side is “female,” and the right side is “male” in Japanese. (**c**) The social cognition task. The left side is “arrogant,” the right side is “panicked,” in Japanese

In the fMRI room, figures were shown on the screen over a mirror set at an angle of 45° in front of the participant’s eyes. We adopted a blocked design to identify brain responses during the social cognition task, with the gender detection task used as the implicit baseline. The alternating epochs of the social cognitive and gender cognition tasks were repeated four times (Fig. 1) using the E-prime software (Psychology Software Tools, Inc., Sharpsburg, PA). A series of figures showing the eye region with different expressions were shown for 6.5 s each, followed by a 0.5-s fixation cross. Each epoch had four pictures and four fixation crosses. The figures were displayed in random order for all participants. The complete sequence of tasks was as follows: (i) six figures of the initial gender detection epoch (42 s); (ii) four figures of the social cognition epoch (28 s); and (iii) four figures of the gender detection epoch (28 s). Epochs (ii) and (iii) were repeated four times, resulting in 133 scans in total. To record responses, participants conducted the fMRI task by pressing the left and right buttons in a controller with four buttons (HHSC-1 × 4-D, CURRENT DESIGNS, Inc., Philadelphia, PA) with their right hand. Following the fMRI measurement, participants were instructed to complete all 36 items of the original RMET questionnaire on paper outside of the MRI room. This was done to allow a comparison of the participants’ social cognitive abilities with scores obtained in previous studies involving healthy individuals and those involving individuals with EDs or ASD. Therefore, we obtained two types of RMET scores: those taken in the MRI room and those taken outside. Additionally, 3D T1 weighted images were acquired for use in the SPM analysis, including co-register and normalization.

### Statistical analyses

An independent t-test was used in IBM SPSS Statistics 28.0 (Armonk, NY: IBM Corp.) for the analysis of demographic and clinical data, the correct response of RMET, and the response time of the fMRI tasks. All data were assessed to determine whether they followed the normal distribution using Levene’s test. Statistical significance was considered to be the 5% level for all variables.

### Data analysis of fMRI

We used the Statistical Parametric Mapping (SPM12; Wellcome Department of Imaging Neuroscience, London [http://www.fil.ion.ucl.ac.uk/spm/]) on MATLAB R2020b (The Mathworks, Inc., Natick, MA, USA) to analyze functional MRI data. After converting the DICOM data to the NifTI format, we completed all preprocessing, including the single subject analysis and the group analysis. During preprocessing, we checked the motion through realignment to exclude participants with a motion greater than 3.0 mm. All resulting images were spatially normalized according to the Montreal Neurological Institute (MNI) template. Spatial smoothing was performed using the Gaussian filter with the full width at half maximum at the kernel size of 8 mm. In addition, a high pass filter of 0.0078 Hz was applied. Second, the design matrix of the social cognition task epoch was set. The first five volumes were cut because of unstable scans in the first 10 scans. We applied the general linear model approach to estimate the parameter on the task. Third, to confirm that the brain regions responsible for recognizing facial expressions were activated more than those for gender judgments, we conducted a one-sample t-test. Moreover, a two-sample t-test was conducted for the whole brain during the tasks for comparing patients and healthy individuals. The following thresholds were applied: *p* < 0.05 with the familywise error (FWE) correction at the peak level and q < 0.05 with the false discovery rate (FDR) correction at the cluster level. Brain maturation has been reported by Javadi et al. [46] to affect the BOLD response to a task; in our study, age was included as a nuisance covariate. Next, we lowered the significance level and analyzed whether there was a trend difference during the social cognition task in BN and HW (*p* < 0.001, uncorrected for multiple comparisons, k > 5 voxels). Fourth, the social cognitive scores obtained during the fMRI were used as a covariate of interest in the correlation analyses with brain responses for both BN and HW, across the whole brain. The threshold in the whole-brain analysis was applied with the following: *p* < 0.05 with the FWE correction at the peak level and q < 0.05 with the FDR correction at the cluster level.

## Results

### Demographic and clinical measures

Table 1 shows the differences in demographic data of the patients and HW. Age, BMI, IQ, and years of education were not significantly different. Responses to clinical measures such as the EDE-Q, BITE, and PHQ-9 revealed significantly more severe conditions in women with BN (*p* < 0.001) than in the HW. There was no significant difference between women with BN and HW, both in the RMET scores in the fMRI (RMET (fMRI)) and on the paper test (RMET (original)) (RMET (fMRI); *p* = 0.21, RMET (original); *p* = 0.69). The mean response time in the social cognition task (mean = 3750.73, SD = 682.03 ms) was significantly longer than in the gender detection task (mean = 1654.68, SD = 378.98 ms) (*p* < 0.001). However, there were no significant differences in reaction time between BN patients and HW in either task (social cognition task, *p* = 0.656; gender detection task, *p* = 0.954). IQ data were collected for 18 BN patients and 22 HW. Response times for the social cognition and gender detection tasks were obtained from 21 BN patients and 22 HW. All other data were collected for 22 BN patients and 22 HW. The IQ data of four patients were missing and one data point for BN was missing.

**Table 1:** Results of the demographic and clinical measures of patients and healthy women

	BN		HW		*p*-value
	Mean	SD	Mean	SD	
Age (years)	26.50	6.79	26.64	7.03	0.948
BMI (kg/m^2^)	19.99	2.02	20.70	1.80	0.228
Duration of illness (years)	5.04	4.97			
IQ	96.69	2.02	99.07	14.55	0.210
Education (years)	14.36	1.85	14.59	1.59	0.664
BITE-SAS	22.59	4.17	5.73	4.21	< 0.001
BITE-SS	11.00	4.98	1.55	1.06	< 0.001
EDE-Q total	3.81	1.32	1.14	0.74	< 0.001
PHQ-9	11.55	5.39	4.36	2.95	< 0.001
GAD-7	7.82	5.11	3.82	2.95	0.003
RMET(original; 36 items)	22.14	3.55	22.55	2.97	0.689
RMET(fMRI; 16 items)	9.14	1.46	8.50	1.82	0.207
Response time of social cognition (ms)	3756.97	658.99	3744.77	718.79	0.656
Response time of gender detection (ms)	1681.48	377.08	1629.11	387.84	0.954
Comorbidities (n)					
Major depressive disorder	5				
Medication (n)					
SSRIs	3				

BN; bulimia nervosa, HW; healthy women, BITE-SAS; the Symptom Assessment Scale of Bulimic Inventory Test, Edinburgh (30), BITE-SS; the Severity Scale of Bulimic Inventory Test, Edinburgh (30), EDE-Q; Eating Disorder Examination Questionnaire (29), PHQ-9; Patient Health Questionnaire - 9 (37), GAD-7; Generalized Anxiety Disorder -7 (38), RMET(fMRI); 16 selected items from the Reading the Mind in the Eyes Test (19) for the fMRI task. RMET(original); The Reading the Mind in the Eyes Test Japanese version on paper. SSRIs; selective serotonin reuptake inhibitors, Student’s t-test: age, BMI, IQ, education, BITE-SAS, RMET (fMRI), RMET (original), and Response time. Welch’s t-test: BITE-SS, EDE-Q total, PHQ-9, and GAD-7. The statistical significance was considered at the 5% level for all variables

### Activation during the social cognition epoch

**Fig. 2:**
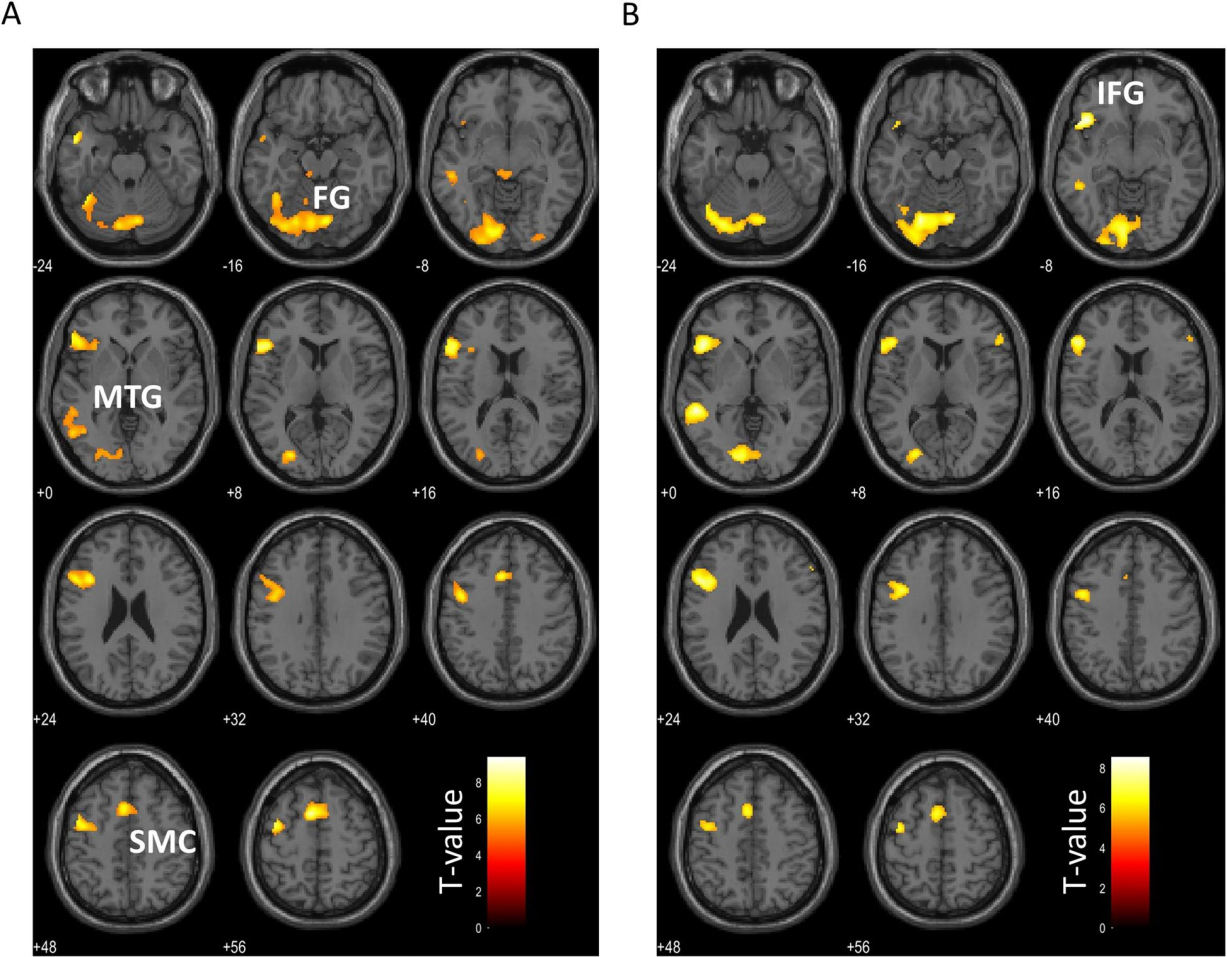
Brain regions with significantly higher BOLD signals during the RMET task in each group as determined by the one-sample t-test (**a**) BN, (**b**) HW, IFG; inferior frontal gyrus, MTG; middle temporal gyrus, FG; fusiform gyrus, SMC; supplementary motor cortex

The brain regions inferior frontal gyrus (IFG), middle temporal gyrus (MTG), superior temporal gyrus (STG), fusiform gyrus, and temporal pole (Table 2) reacted more during the social cognition epoch than during the gender detection epoch (Fig. 2). The mPFC, temporoparietal junction (TPJ), posterior superior temporal sulcus (pSTS), posterior cingulate cortex (PCC), precuneus, and temporal pole coincided with the brain regions reported to be associated with mentalizing [47–49].

**Table 2:** Brain regions with more activation in the social cognition epoch than in the gender detection epoch

Region	Hemisphere	Voxels	T-score	MNI coordinates		
				x	y	z
BN						
Triangular part of inferior frontal gyrus, Opercular part of the inferior frontal gyrus	L	1847	9.39	−50	24	4
Supplementary motor cortex	L	734	8.84	−6	10	59
Temporal pole, Middle temporal gyrus	L	115	8.02	−50	2	−22
Fusiform gyrus, Inferior temporal gyrus	L	2812	7.95	−40	−54	−22
Middle temporal gyrus, Superior temporal gyrus	L	447	7.80	−52	−30	−4
Occipital fusiform gyrus, Inferior occipital gyrus	R	111	6.46	32	−88	−12
Brain stem, L/R Ventral diencephalon	–	105	6.17	0	−30	−10
HW						
Triangular part of the Inferior frontal gyrus, Frontal opeculum	L	1816	8.54	−46	26	4
Middle temporal gyrus, Superior temporal gyrus	L	431	8.54	−52	−40	−2
Occipital fusiform gyrus, Lingual gyrus	L	2868	7.94	−20	−74	−16
Supplementary motor cortex	L	400	7.05	−6	8	54
Triangular part of the Inferior frontal gyrus, Opercular part of the inferior frontal gyrus	R	104	6.56	52	26	4
Occipital fusiform gyrus, Occipital pole	R	51	6.03	26	−92	−12

Height threshold: *p* < 0.05, familywise error (FWE) for multiple comparisons; cluster threshold: *p* < 0.05, false discovery rate (FDR) corrected for multiple comparisons

A one-sample t-test was conducted. *MNI* Montreal Neurological Institute, *BN* women with bulimia nervosa, *HW* healthy women, *R* Right, *L* Left

### Difference in activation between BN patients and HW

The activation of BOLD signals reacting to the social cognition epoch was not significantly different between women with BN and HW (*p* < 0.05). However, when the same analysis was performed with reduced significance levels (*p* < 0.001 (unc.), k > 5 voxels), the angular gyrus, ventral diencephalon, thalamus proper, temporal pole, and middle temporal pole showed a more robust BOLD signal change during the social cognition epoch in patients with BN compared to HW. Table 3 shows the activated brain regions of the social cognition epoch on the threshold uncorrected *p* < 0.001 at the peak level.

**Table 3:** Brain regions with more activation in the social cognition epoch than in the gender detection epoch in patients versus healthy women

Region	Hemisphere	Voxels	T-score	MNI coordinates		
				x	y	z
BN > HW						
Angular gyrus	R	22	3.80	40	−56	34
Ventral diencephalon, Thalamus proper	R	10	3.63	10	−16	−8
Temporal pole, Middle temporal gyrus	L	6	3.32	−48	4	−24
BN < HW						
None						

Height threshold: *p* < 0.001, uncorrected; cluster threshold: k > 5 voxels. A Two sample t-test was adopted. *MNI* Montreal Neurological Institute, *BN* women with bulimia nervosa, *HW* healthy women, *R* Right, *L* Left

### Correlation with the BOLD signals and the number of correct answers on the RMET

**Fig. 3:**
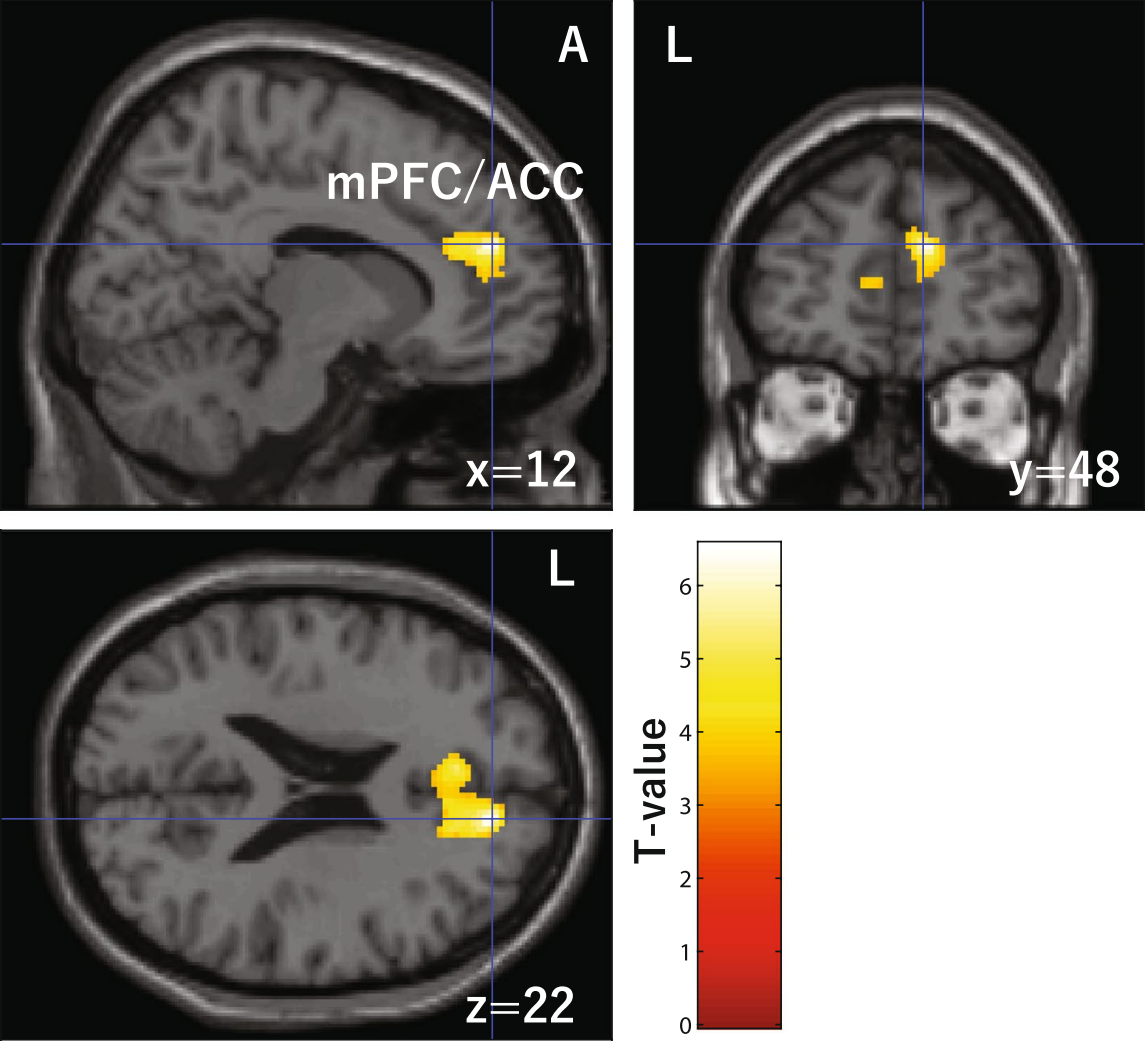
One cluster that has a positive correlation with the score of RMET in the social cognition task mPFC, medial prefrontal cortex; ACC, anterior cingulate cortex; A, anterior; L, left

There was a positive correlation between the activation of BOLD signals in some brain regions and the number of correct answers on the RMET in HW. No such correlation was observed in women with BN (Fig. 3). The MNI coordinates shown in Table 4 mainly belonged to the medial prefrontal cortex (mPFC; Brodmann area 9), dorsal anterior cingulate cortex (dACC; Brodmann area 32). There was no negative correlation between these regions in the HW. No positive or negative correlations between the social cognition phase and brain regions were observed in BN patients.

**Table 4:** Brain regions correlated with RMET scores and activation of BOLD signals during the social cognition task for healthy women

Regions	Hemisphere	Voxels	T-score	MNI coordinates		
				x	y	z
Positive						
Superior frontal gyrus, anterior cingulate gyrus, medial frontal gyrus, superior frontal gyrus	R	851	6.55	12	48	22
			5.50	−6	36	20
			5.18	14	36	20
Negative						
None						

Height threshold: *p* < 0.001, uncorrected for multiple comparisons; cluster threshold: *p* < 0.05, false discovery rate (FDR) corrected for multiple comparisons. Positive; the positive correlation between the BOLD signals and RMET score in healthy women, Negative; the negative correlation between the BOLD signals and RMET score in healthy women, *MNI* Montreal Neurological Institute, *R* Right, *L* Left

## Discussion

We examined the differences in neural responses related to social cognition between patients with BN and HW. In HW, BOLD responses to the RMET task in the mPFC and dACC correlated positively to the scores of the social cognition epoch of the RMET. However, no correlated neural responses were found in women with BN. These results suggest the possibility of estimating the emotions of others with a circuit that does not rely on mPFC and dACC in BN patients.

The results on the social cognition of BN patients in previous studies are controversial. Although one study showed that BN patients had poor RMET scores compared to controls [20], another reported that the total RMET score was not significantly different between BN and HW, while there was a difference between women with AN and HW [50]. In our study, there was no significant difference in the scores of social cognition tasks, the original RMET on paper, or the RMET epoch during the fMRI scan, between patients and HW. This implies that BN patients may not have a deficit of social cognition as implied by the RMET, although we cannot deny the possibility that our task was not sufficiently acute for measuring their abilities. For example, Black et al. [51] claimed that the social cognitive RMET task is insufficient in measuring the social cognition ability of neurotypical adults. This may be one reason why there were no differences in the social cognition task scores between patients and HW.

From comparisons of activation during the RMET task in both HW and patients with BN, social brain areas such as IFG, MTG, STG, fusiform gyrus, and temporal pole were activated more significantly in the social cognition epoch than the gender detection epoch, which supports previous studies [48, 49, 52] (Table 2). The results of the social cognition task we used reflected the brain responses when thinking about others’ mental states. Therefore, even though the task we adopted was not sufficiently acute, it was still appropriate as a social cognition task. In addition, the correlation analysis of BOLD responses and RMET scores demonstrates that the more the healthier women answered correctly during the social cognition epoch, the more the mPFC and dACC were activated (Table 3). This was not observed in women with BN (Fig. 2). The mPFC is one of the regions related to the theory of mind (ToM), an important aspect of social cognition [53]. Zeng et al. [53] reported that their Brain-ToM model raised STS, TPJ, inferior parietal lobule (IPL), pSTS, PCC, ACC, mPFC, ventral medial prefrontal cortex, dorsal medial prefrontal cortex, IFG, ventral premotor cortex, and primary motor cortex. Among patients with AN, less activation was shown on the social cognition task in the mPFC, STS, and temporal pole compared to HW [13]. Concerning emotional cognition, the mentalizing system (also ToM network) consists of the mPFC, precuneus, PCC, amygdala, temporoparietal junction, and temporal pole [54]. Furthermore, in a task-based fMRI study regarding irony comprehension, the activity of the mPFC was decreased in children with ASD who have severely impaired social skills [55]. In our results the mPFC response and the emotional task scores were positively correlated in HW. However, no significant correlation between the RMET score and mPFC activity was found in BN patients. The mPFC plays an important role in two neural systems: the mirror neuron system and the mentalizing system (the ToM network) [54, 55]. This result is consistent with that of studies showing decreased mPFC activity in people with AN and ASD [13, 55]. Therefore, these three disorders (BN, AN, and ASD) have a common feature—the decrease in mPFC activation indicating a deficit of social cognition.

Nevertheless, we cannot exclude the possibility that the social cognitive abilities of BN patients are preserved. This is because there was no significant difference in RMET scores. From the results of the two-sample t-test, although no significant group differences were found between BN and HW, a closer examination of the sites of significant BOLD signal change in BN and HW (Table 3) revealed an increase in BOLD signal response during the task in the right angular gyrus, ventral diencephalon and thalamus proper, left temporal pole and middle temporal gyrus in the BN compared to the HW. In connection with the finding that the angular gyrus is involved in semantic memory [56], in our study, the participants chose one of the complex options for the emotion around the eyes, suggesting that the BN patients were more likely than the HW to carefully consider the content of the options and to use a strategy to elicit responses. However, the response time during the social cognition task in BN patients was about the same as in HW. Therefore, it is unclear whether BN patients use strategies that rely on semantic memory in situations requiring social cognition. In addition, Ruan et al. [57] reported that hyperactivation of the temporal pole and middle temporal gyrus was observed during social cognition tasks in BN compared to healthy controls. The authors claim that this result contrasts with those of other studies that have shown reduced temporal pole activity in AN compared to healthy controls, which was also observed in our study. In addition, the activity in thalamus was detected more robustly in the BN during the social cognition task (Table 3). The thalamus is reported to be connected to the cortex and to control various forms of cognition [59]. It is possible that social cognition in BN relies on distinct circuits compared to healthy individuals, where the thalamus coordinates the angular gyrus, temporal pole, and middle temporal gyrus.

Our study can be summarized as follows. While we could not clarify the group differences on the social cognition task, the correlations among BN and HW on the mPFC and dACC (which are related to social cognition) differed. The insensitivity of the RMET for neurotypical adults [51] might have resulted in the absence of a difference between BN patients and HW on the social cognition task. While the mPFC plays important roles in social cognition [53, 54], we identified the deviation of BN patients from HW in some brain regions. Further studies are necessary to investigate the features of BN patients using a task that is able to measure the dysfunction of social cognition more accurately.

### Limitations

This study has five limitations. First, our patient and HW sample sizes of were not large enough. With a larger sample size, more detailed analyses could have been conducted. Second, medication was not controlled, and there were missing values for the medication of two patients. The patients’ medication may have affected their brain function, which in turn, may have affected the results of the study. Third, the comorbidity of patients was also not controlled. Such factors may have influenced the activation of the brain. Fourth, participants responded to the modified RMET inside the MRI and then responded to the original version of the RMET on paper outside the MRI room. Although we did not communicate the answers after the task was completed in the MRI room, we performed the RMET twice—both inside and outside the MRI room (16 and 36 items, respectively)—which may have had a learning effect on the scores of the paper test. Fifth, the IQ was scored using WAIS-III. Any variations from the latest version may have impacted the accuracy of IQ measurements.

## Conclusions

We examined differences in the brain regions activated during a social cognition task given to BN and HW. In HW, BOLD responses in the mPFC and dACC were positively correlated with the RMET social cognition epoch scores. However, none of these brain responses were observed in women with BN. These results suggest that BN, as with AN, modulates brain activities when thinking about others’ mental states. More research is needed to reveal the neural processes of social cognition in people with BN.

## Data Availability

Not applicable.

## Abbreviations

ACC: anterior cingulate cortex
AN: anorexia nervosa
ASD: autism spectrum disorder
BED: binge-eating disorder
BITE: Bulimic Investigatory Test, Edinburgh
BMI: body mass index
BN: bulimia nervosa
BOLD: blood-oxygenation-level dependent
dACC: dorsal anterior cingulate cortex
ED: eating disorder
EDE-Q: Eating Disorders Examination Questionnaire
FDR: false discovery rate
fMRI: functional magnetic resonance imaging
FWE: familywise error
GAD-7: Generalized Anxiety Disorder
HW: healthy women
IFG: inferior frontal gyrus
IPL: inferior parietal lobule
IQ: intelligence quotient
MNI: Montreal Neurological Institute
mPFC: medial prefrontal cortex
MTG: middle temporal gyrus
PCC: posterior cingulate cortex
PHQ-9: Patient Health Questionnaire
pSTS: posterior superior temporal sulcus
RMET: Reading Mind in the Eyes Test
SS: Severity Scale
STG: superior temporal gyrus
SAS: Symptom Assessment Scale
ToM: theory of mind
TPJ: temporoparietal junction
WAIS-III: Wechsler Adult Intelligence Scale, third edition
YLD: years lived with a disability

## References

[1] Diagnostic and Statistical Manual of Mental Disorders. 5th ed. Arlington: American Psychiatric Association; 2013.

[2] van Eeden AE, van Hoeken D, Hoek HW (2021). Incidence, prevalence and mortality of anorexia nervosa and bulimia nervosa. Curr Opin Psychiatry..

[3] Mele G, Alfano V, Cotugno A, Longarzo M (2020). A broad-spectrum review on multimodal neuroimaging in bulimia nervosa and binge eating disorder. Appetite..

[4] Arcelus J, Mitchell AJ, Wales J, Nielsen S (2011). Mortality rates in patients with anorexia nervosa and other eating disorders. A meta-analysis of 36 studies. Arch Gen Psychiatry..

[5] Hoeken van D, Hoek HW (2020). Review of the burden of eating disorders: mortality, disability, costs, quality of life, and family burden. Curr Opin Psychiatry..

[6] GBD. Disease and injury incidence and prevalence collaborators. Global, regional, and national incidence, prevalence, and years lived with disability for 354 diseases and injuries for 195 countries and territories, 1990–2017: a systematic analysis for the global burden of disease study 2017. Lancet. 2018:1789–858. 10.1016/S0140-6736(18)32279-7. 10.1016/S0140-6736(18)32279-7 PMC6227754 30496104

[7] Fairburn CG, Cooper Z, Shafran R, Wilson GT, Barlow DH (2008). Eating disorders: a transdiagnostic protocol. Clinical handbook of psychological disorders: a step-by-step treatment manual.

[8] Baker CL, Jara-Ettinger J, Saxe R, Tenenbaum JB (2017). Rational quantitative attribution of beliefs, desires and percepts in human mentalizing. Nat Hum Behav..

[9] Happé F, Cook JL, Bird G. The structure of social cognition: in (ter) dependence of sociocognitive processes. Annu Rev Psychol. 2017(68):243–67. 10.1146/annurev-psych-010416-044046. 10.1146/annurev-psych-010416-044046 27687121

[10] Tauro JL, Wearne TA, Belevski B, Filipčíková F, Francis HM. Social cognition in female adults with anorexia nervosa: a systematic review. Neurosci Biobehav Rev. 2022(132):197–210. 10.1016/j.neubiorev.2021.11.035. 10.1016/j.neubiorev.2021.11.035 34822877

[11] Baron-Cohen S, Leslie AM, Frith U (1985). Does the autistic child have a “theory of mind”?. Cognition..

[12] Hamatani S, Tomotake M, Takeda T, Kameoka N, Kawabata M, Kubo H, et al. Impaired social cognition in anorexia nervosa patients. Neuropsychiatr Dis Treat. 2016:2527–31. 10.2147/NDT.S116521. 10.2147/NDT.S116521 PMC5063555 27785029

[13] Brockmeyer T, Pellegrino J, Münch H, Herzog W, Dziobek I, Friederich HC (2016). Social cognition in anorexia nervosa: specific difficulties in decoding emotional but not nonemotional mental states. Int J Eat Disord..

[14] Schulte-Rüther M, Mainz V, Fink GR, Herpertz-Dahlmann B, Konrad K (2012). Theory of mind and the brain in anorexia nervosa: relation to treatment outcome. J Am Acad Child Adolesc Psychiatry..

[15] Mason TB, Lesser EL, Dolgon-Krutolow AR, Wonderlich SA, Smith KE. An updated transdiagnostic review of social cognition and eating disorder psychopathology. J Psychiatr Res. 2021(143):602–27. 10.1016/j.jpsychires.2020.11.019. 10.1016/j.jpsychires.2020.11.019 33190838

[16] Kerr-Gaffney J, Harrison A, Tchanturia K (2019). Cognitive and affective empathy in eating disorders: a systematic review and meta-analysis. Front Psychiatry..

[17] Dejong H, den Eynde FV, Broadbent H, Kenyon MD (2013). A lavender, H startup, U Schmidt. Social cognition in bulimia nervosa: a systematic review. Eur Psychiatry..

[18] Dapelo MM, Urguladze S, Morris R, Tchanturia K (2017). Emotion recognition in face and body motion in bulimia nervosa. Eur Eat Disord Rev..

[19] Baron-Cohen S, Wheelwright S, Hill J, Raste Y, Plumb I (2001). The “Reading the Mind in the Eyes” Test revised version: A study with normal adults, and adults with Asperger syndrome or high-functioning autism. J Child Psychol Psychiatry..

[20] Sanislow CA, Ferrante M, Pacheco J, Rudorfer MV, Morris SE (2019). Advancing translational research using NIMH research domain criteria and computational methods. Neuron..

[21] Preti A, Siddi S, Marzola E, Abbate Daga GA. Affective cognition in eating disorders: a systematic review and meta-analysis of the performance on the “Reading the mind in the eyes”. Test Eat Weight Disord. 2022:2291–307. 10.1007/s40519-022-01393-8. 10.1007/s40519-022-01393-8 PMC9556412 35384555

[22] Serra R, Di Nicolantonio C, Di Febo R, De Crescenzo F, Vanderlinden J, Vrieze E, et al. The transition from restrictive anorexia nervosa to binging and purging: a systematic review and meta-analysis. Eat Weight Disord. 2022(27):857–65. 10.1007/s40519-021-01226-0. 10.1007/s40519-021-01226-0 PMC8964622 34091875

[23] Corsi E, Cardi V, Sowden S, Coll MP, Cascino G, Ricca V (2021). Socio-cognitive processing in people with eating disorders: computerized tests of mentalizing, empathy and imitation skills. Int J Eat Disord..

[24] Zanella E, Lee E (2022). Integrative review on psychological and social risk and prevention factors of eating disorders. Heliyon..

[25] Tuschen-Caffier B, Vögele C (1999). Psychological and physiological reactivity to stress: an experimental study on bulimic patients, restrained eaters and controls. Psychother Psychosom..

[26] Hinrichsen H, Wright F, Waller G, Meyer C (2003). Social anxiety and coping strategies in eating disorders. Eat Behav..

[27] Fairburn CG, Stice E, Cooper Z, Doll HA, Norman PA, O'Connor ME (2003). Understanding persistence in bulimia nervosa: a 5-year naturalistic study. J Consult Clin Psychol..

[28] American Psychological Association (2002). Diagnostic and statistical manual of mental disorders.

[29] Fairburn CG, Beglin SJ. Assessment of eating disorders: interview or self-report questionnaire? Int J Eat Disord. 1994;16:363–70. 7866415

[30] Henderson M, Freeman CP (1987). A self-rating scale for bulimia. The “BITE”. Br J Psychiatry..

[31] Nagata JM, Compte EJ, Murray SB, Schauer R, Pak E, Flentje A (2021). Community norms for the eating disorder examination questionnaire (EDE-Q) among cisgender bisexual plus women and men. Eat Weight Disord..

[32] Luce KH, Crowther JH, Pole M (2008). Eating disorder examination questionnaire (EDE-Q): norms for undergraduate women. Int J Eat Disord..

[33] Aardoom JJ, Dingemans AE, Landt MCTSO, Furth EFV (2012). Norms and discriminative validity of the eating disorder examination questionnaire (EDE-Q). Eat Behav..

[34] Streiner DL, Norman GR, Cairney J (2015). Health measurement scales: a practical guide to their development and use.

[35] Melisse B, Furth EFV, de Beurs E (2022). Eating disorder examination questionnaire (EDE-Q): validity and norms for Saudi nationals. Eat Weight Disord..

[36] Kroenke K, Spitzer RL, Williams JB (2001). The PHQ-9: validity of a brief depression severity measure. J Gen Intern Med..

[37] Spitzer RL, Kroenke K, Williams JBW, Löwe B (2006). A brief measure for assessing generalized anxiety disorder: the GAD-7. Arch Intern Med..

[38] Muramatsu K, Miyaoka H, Kamijima K, Muramatsu Y, Tanaka Y, Hosaka M (2018). Performance of the Japanese version of the patient health Questionnaire-9 (J-PHQ-9) for depression in primary care. Gen Hosp Psychiatry..

[39] Martin A, Rief W, Klaiberg A, Braehler E (2006). Validity of the brief patient health questionnaire mood scale (PHQ-9) in the general population. Gen Hosp Psychiatry..

[40] Löwe B, Decker O, Müller S, Brähler E, Schellberg D, Herzog W (2008). Validation and standardization of the generalized anxiety disorder screener (GAD-7) in the general population. Med Care..

[41] Dhira TA, Rahman MA, Sarker AR, Mehareen J. Validity and reliability of the generalized anxiety Disorder-7 (GAD-7) among university students of Bangladesh. PLoS One. 2021;16(12) 10.1371/journal.pone.0261590. 10.1371/journal.pone.0261590 PMC8675645 34914811

[42] Pavlova MA, Sokolov AA (2022). Reading the language of the eyes. Neurosci Biobehav Rev..

[43] Wechsler D (1997). Wechsler Adult Intelligence Scale.

[44] Sumiyoshi C, Fujino H, Sumiyoshi T, Yasuda Y, Yamamori H, Ohi K (2016). Usefulness of the Wechsler intelligence scale short form for assessing functional outcomes in patients with schizophrenia. Psychiatry Res..

[45] Rolls ET, Grabenhorst F, Deco G. Decision-making, errors, and confidence in the brain. J Neurophysiol. 2010;104(5):2359-74. 10.1152/jn.00571.2010. Epub 2010 Sep 1. 10.1152/jn.00571.2010 20810685

[46] Javadi AH, Schmidt DHK, Smolka MN (2014). Differential representation of feedback and decision in adolescents and adults. Neuropsychologia..

[47] Frith CD, Frith U (2006). The neural basis of mentalizing. Neuron..

[48] Carrington SJ, Bailey AJ (2009). Are there theory of mind regions in the brain? A review of the neuroimaging literature. Hum Brain Mapp..

[49] Tapajóz P, de Sampaio F, Soneira S, Aulicino A, Martese G, Iturry M, et al. Theory of mind and central coherence in eating disorders: two sides of the same coin? Psychiatry Res. 2013;(210):1116–22. 10.1016/j.psychres.2013.08.051. 10.1016/j.psychres.2013.08.051 24064463

[50] Black JE. An IRT analysis of the reading the mind in the eyes test. J Pers Assess. 2019(101):425–33. 10.1080/00223891.2018.1447946. 10.1080/00223891.2018.1447946 29611711

[51] Schurz M, Radua J, Aichhorn M, Richlan F, Perner J. Fractionating theory of mind: a meta-analysis of functional brain imaging studies. Neurosci Biobehav Rev. 2014;(42):9–34. 10.1016/j.neubiorev.2014.01.009. 10.1016/j.neubiorev.2014.01.009 24486722

[52] Zeng Y, Zhao Y, Zhang T, Zhao D, Zhao F, Lu E. A brain-inspired model of theory of mind. Front Neurorobot. 2020; 10.3389/fnbot.2020.00060. 10.3389/fnbot.2020.00060 PMC7483660 32982714

[53] Vogeley K (2017). Two social brains: neural mechanisms of intersubjectivity. Philos Trans R Soc Lond Ser B Biol Sci..

[54] Wang AT, Lee SS, Sigman M, Dapretto M (2007). Reading affect in the face and voice: neural correlates of interpreting communicative intent in children and adolescents with autism spectrum disorders. Arch Gen Psychiatry..

[55] Humphreys GF, Ralph MAL, Simons JS (2021). A unifying account of angular gyrus contributions to episodic and semantic cognition. Neurosci..

[56] Ruan VA, Hartz A, Hueck M, Dahmen B, Polier G, Herpertz-Dahlmann B (2022). Neural mechanisms underlying social recognition and theory of mind in adolescent patients with bulimia nervosa and transdiagnostic comparison with anorexia nervosa. Eur Eat Disord Rev..

[57] Pergola G, Danet L, Pitel AL, Carlesimo GA, Segobin S, Pariente J (2018). The regulatory role of the human mediodorsal thalamus. Trends Cogn Sci..

